# Monitoring levetiracetam concentration in saliva during pregnancy is stable and feasible

**DOI:** 10.1111/cns.14827

**Published:** 2024-07-11

**Authors:** Wanling Li, Ximeng Yang, Qian Chen, Zhenlei Wang, Yifei Duan, Lei Chen

**Affiliations:** ^1^ Department of Neurology, West China Hospital Sichuan University Chengdu Sichuan China; ^2^ Center of Biostatistics, Design, Measurement and Evaluation, Department of Clinical Research Management West China Hospital of Sichuan University Chengdu Sichuan China; ^3^ NMPA Key Laboratory for Clinical Research and Evaluation of Innovative Drug, Clinical Trial Center West China Hospital of Sichuan University Chengdu Sichuan China; ^4^ Department of Clinical Research Management, West China Hospital Sichuan University Chengdu Sichuan China

**Keywords:** levetiracetam, plasma, pregnant women with epilepsy, saliva, therapeutic drug monitoring

## Abstract

**Aims:**

This multicenter prospective cohort study (registration no. ChiCTR2000032089) aimed to investigate the relationship between saliva and plasma levetiracetam concentrations to determine whether saliva could be used for routine monitoring of levetiracetam during pregnancy.

**Methods:**

The slot concentrations of levetiracetam in simultaneously obtained saliva and plasma samples were measured using UPLC–MS/MS. The correlations between saliva and plasma levetiracetam concentrations and the dose‐normalized concentrations were compared among pregnant women in different stages and nonpregnant control participants with epilepsy.

**Results:**

In total, 231 patients with 407 plasma and saliva sample pairs were enrolled from 39 centers. Linear relationships between salivary and plasma levetiracetam concentrations were reported in the enrolled population (*r* = 0.898, *p* < 0.001), including pregnant (*r* = 0.935, *p* < 0.001) and nonpregnant participants (*r* = 0.882, *p* < 0.001). Plasma concentrations were moderately higher than saliva concentrations, with ratios of saliva to plasma concentrations of 0.98 for nonpregnant women, 0.98, 1, and 1.12 for pregnant women during the first trimester, the second trimester, the and third trimester, respectively. The effective range of saliva levetiracetam concentration was found to be 9.98 μg/mL (lower limit) with an area under the curve (AUC) of 0.937 (95% confidence intervals, 0.915–0.959), sensitivity of 88.9%, specificity of 86.8%, and *p* < 0.001, to 24.05 μg/mL (upper limit) with an AUC of 0.952 (0.914–0.99), sensitivity of 100%, specificity of 92.3%, and *p* = 0.007.

**Conclusion:**

The saliva/plasma concentration ratio of levetiracetam remains constant during pregnancy and is similar to that in non‐pregnant individuals. Monitoring levetiracetam concentration in saliva during pregnancy should be widely promoted.

## Graphical abstract

Salivary and plasma levetiracetam concentrations had a linear relationship and similar changes during pregnancy, which supported the effective use of saliva in levetiracetam therapeutic drug monitoring during pregnancy.

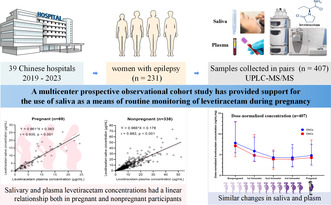

## 1 INTRODUCTION

Epilepsy, a common chronic neurological disorder, is mainly treated by antiseizure medications (ASMs)^1^; Levetiracetam is one of the most commonly prescribed second‐generation ASMs in women with epilepsy (WWE) during pregnancy.^2^, ^3^, ^4^, ^5^, ^6^ Pregnancy‐induced pharmacokinetic changes, including decreased absorption, distribution changes, and increased clearances, result in a 36.8% dose‐normalized levetiracetam concentration decline during pregnancy, significantly increasing the risk of seizures.^3^, ^7^, ^8^, ^9^, ^10^ Management of epilepsy in pregnancy requires balancing risk of harm from suboptimal therapy and worsening maternal seizures with fetal risk from increased levetiracetam exposure due to unnecessary dose increases.^11^, ^12^

Guidelines recommend monitoring levetiracetam concentration monthly^3^ and adjusting the dosage based on the monitoring results to reduce the incidence of stillbirth and fetal malformation caused by levetiracetam overdose, as well as to reduce the maternal mortality rate caused by worsening seizures due to insufficient dosage.^13^ Therapeutic drug monitoring (TDM) can guide the adjustment of levetiracetam dosage to optimize clinical outcomes and help confirm medication adherence.^11^, ^13^, ^14^ However, routine TDM performed with invasive blood samples may cause discomfort to pregnant women, resulting in poor acceptance.^3^, ^9^, ^15^ Conversely, the use of saliva for levetiracetam monitoring during pregnancy has the advantages of convenient non‐invasive sampling, unrestricted sampling sites, small amounts of saliva required, and low‐storage requirements.^16^, ^17^, ^18^ Levetiracetam enters saliva by passive diffusion, and it is concentration in saliva reflect the non‐protein bound free concentration (pharmacologically active) in plasma.^19^

Saliva and plasma levetiracetam concentrations have been reported in the general population with limited sample size;^19^, ^20^, ^21^, ^22^, ^23^ and to our knowledge, no prospective study has been performed focusing on pregnant women with epilepsy. To provide additional evidence, this study aims to characterize pregnancy‐associated concentration changes and to formally investigate the relationship between saliva and plasma levetiracetam concentrations in different gestational stages among the Chinese population, and to determine whether saliva can be used for routine monitoring of levetiracetam during pregnancy.

## 2 METHODS

### 2.1 Patient population

The multicenter prospective observational cohort study was approved by the medical ethics committee of each study site and the Institutional Review Board of West China Hospital of Sichuan University (Approval IRB No. 2019‐870; Chengdu, China), and registered in Chinese Clinical Trial Registry (www.chictr.org.cn, number ChiCTR2000032089). Informed written consent was obtained from all participants or their legal guardians. This study followed the Strengthening the Standards for Reporting of Diagnostic Accuracy (STARD).

Women aged 14–45 years, diagnosed with epilepsy at 39 Chinese hospitals from October 2019 to March 2023, receiving dose‐stabilized levetiracetam for more than 3 months, were included in this study. Exclusion criteria included exposure to interacting medications, physical examination confirmed comorbidities affecting the levetiracetam absorption, and progressive cerebral disease. Epilepsy was diagnosed by two experienced neurologists in accordance with the International Statistical Classification of Diseases and Related Health Problems (ICD‐10).^24^ We investigated clinical information including basic demographic characteristics, medical history, and levetiracetam doses. Seizure frequency was based on a seizure diary covering the 3 months prior to sampling. Gestational age (GA) was determined based on the last menstrual period. The levetiracetam concentrations were then classified as nonpregnant (not pregnant or before pregnancy) and pregnant, including the first trimester (≤13 weeks GA), the second trimester (14–27 weeks GA), or the third trimester (≥28 weeks GA—delivery).^5^ The gold standard for establishing a reference concentration range for saliva was the reference effective concentration range of levetiracetam in plasma, which ranged from 12 to 46 μg/mL.^7^, ^25^ Dose‐normalized concentrations (DNCs) were calculated as saliva or plasma levetiracetam concentrations divided by total daily dose.^7^

### 2.2 Plasma and saliva samples collection, preparation, and storage

Blood and saliva samples were collected simultaneously (within 15 min), prior to the first morning dose to determine fasting values (trough steady‐state concentration) and 1 h after the morning administration of levetiracetam. The mouth was rinsed with water 15 min prior to saliva collection, and then approximately 2–3 mL of unstimulated saliva was collected using a Salivette devices (Sarstedt, Nümbrecht, Germany)^26^ and centrifuged at 1600×*g* for 15 min at room temperature. At least 5 mL of venous whole‐blood was collected in an ethylenediaminetetraacetic acid (EDTA) tube and plasma was separated from blood cells by centrifugation for 15 min (4°C, 1600×*g*). Within 30 min after sampling, plasma and saliva samples were stored at −80°C until analysis.

### 2.3 Drug concentrations detection and analysis

The concentrations of drugs were measured via validated ultra performance liquid chromatography (Agilent Technologies) coupled with tandem mass spectrometry (QTRAP 5500, Applied Biosystems) (UPLC–MS/MS) at West China Hospital of Sichuan University in the Clinical Trial Center/NMPA Key Laboratory for Clinical Research and Evaluation of Innovative Drug. The levetiracetam measured and quantification ranges were 0.1–50 μg/mL in both plasma and saliva samples.

Plasma and saliva samples were thawed to room temperature and 40 μL were dispensed into 1.5‐mL polypropylene microcentrifuge tubes containing 760 μL of acetonitrile (Thermo Fisher, LC‐MS‐grade) with internal standard (IS) (0.02 mg/L, Levetiracetam‐d6, purity ≥98%), vortexed for 1 min, and then centrifuged for 5 min (4°C, 13,000 rpm). The 25 μL organic supernatant was transferred to a clean polypropylene tube and reconstituted with 975 μL of mobile phase A. The mixture was shaken on a vibrax mixer for 1 min. The extract was transferred to an autosampler vial and 7.5 μL was injected into the UPLC‐MS/MS system.

The MS/MS unit was conducted in positive electrospray ionization mode and was used for mass spectrometry detection. Waters Acquity BEH C18 column (2.1 × 50 mm i.d., 1.7 mm; Waters Co., Milford, MA, USA) was used for chromatographic separation under isocratic conditions, and the column temperature was maintained at 40°C. Mobile phase A consisted of 0.1% formic acid and 5 mmol/L ammonium acetate aqueous solution, and mobile phase B consisted of acetonitrile/methanol/formic acid (500/500/1, vol/vol/vol) at a flow rate of 0.4 mL/min. Sample analysis was performed in multiple reaction monitoring mode with m/z values of 171.0 → 126.1 for levetiracetam and 177 → 131.1 for levetiracetam‐d6, respectively.

All validation experiments, including sensitivity, specificity, precision, accuracy, recovery, matrix effects, and method stability, were conducted according to the current US Food and Drug Administration (FDA), European Medicines Agency (EMA), and NMPA guidelines for validation of bioanalytical methods.^27^

### 2.4 Statistical analysis

Women taking levetiracetam monotherapy or combination therapy with other non‐interacting antiepileptic drugs were considered for this analysis. To account for varied and changing doses, we used DNCs in all analyses: DNC = levetiracetam concentration (μg/mL)/levetiracetam total daily dose (mg). All data were analyzed by SPSS 26.0 (IBM), and all figures were created using Prism. The normal distribution of the assay results was evaluated by Shapiro–Wilk test. Results were presented as means ± SD unless otherwise specified. Student's *t*‐test was used to compare differences between the two groups and Analysis of variance (ANOVA) was used to compare differences among multiple groups. Correlation analysis was performed using Pearson correlation coefficients for concentrations in different matrices. The degree of agreement between plasma and saliva concentrations was also assessed by Pearson linear regression, and 95% confidence intervals were calculated for the slope and intercept of the regression line. The reference concentration range for levetiracetam in saliva was established by plotting the Receiver Operating Characteristic (ROC) curve. A *p* < 0.05 was considered statistically significant.

## 3 RESULTS

### 3.1 Patient characteristics and levetiracetam concentrations

A total of 231 patients (median [range] age, 27 [14–45] years) were enrolled, of whom 97 patients provided samples more than one time, and demographic characteristics (age, weight, and height) of the pregnant and control women were similar (Table 1). Specifically, 407 plasma and 407 saliva samples collected in pairs (morning samples and samples after intake of levetiracetam) were tested. Patients had median daily doses of 1000 (125–3000) mg in the nonpregnant group and 1000 (250–2750) mg in the pregnant group without significant difference. The mean concentrations of levetiracetam were 10.86 μg/mL in plasma and 10.69 μg/mL in saliva (Table 1). During pregnancy, the concentration of levetiracetam in both plasma and saliva was lower than in non‐pregnant individuals (*p* < 0.001). Although there was no statistically significant difference in seizure frequency in the last/3 months prior to sampling between non‐pregnant and pregnant individuals, there was a trend of increased seizure counts during pregnancy compared to the non‐pregnant period, with the second trimester having the highest frequency of seizures (Table 1). Additionally, there was no significant correlation between LEV plasma/saliva levels and the number of seizures in the previous 1/3 months (*p* > 0.05).

**TABLE 1:** Demographic characteristics of the cohort of pregnant and control nonpregnant women with epilepsy taking levetiracetam (LEV).

Cohort variable	Total	Nonpregnant	Pregnant		First trimester		Second trimester		Third trimester	
	Mean ± SD	Mean ± SD	Mean ± SD	*p*‐value	Mean ± SD	*p*‐value	Mean ± SD	*p*‐value	Mean ± SD	*p*‐value
Patients, no.	231	200	31		14		12		5	
Age, years	27.92 ± 6.18	27.99 ± 6.42	27.52 ± 4.43	0.695	26.07 ± 2.95	0.27	29.33 ± 5.11	0.476	27.2 ± 5.45	0.787
Weight, kg	55.48 ± 8.97	55.25 ± 9.11	56.95 ± 8.04	0.329	54.84 ± 8.49	0.872	57.33 ± 7.22	0.438	61.9 ± 7.8	0.107
Height, cm	160.1 ± 5.25	160 ± 5.26	161 ± 5.19	0.359	160.8 ± 5.51	0.635	161.6 ± 5.82	0.317	160 ± 3.08	0.999
All (morning samples and samples after intaking of LEV) saliva and plasma samples collected in pairs										
Sample pairs, no.	407	338	69		30		25		14	
LEV daily dose, mg	1227.8 ± 501	1232 ± 498.6	1206.5 ± 516	0.7	1075 ± 342.1	0.026	1290 ± 623.7	0.583	1339.3 ± 585	0.434
LEV plasma concentration, μg/mL	10.86 ± 8.45	11.67 ± 8.72	6.86 ± 5.47	<0.001	7.46 ± 5.66	0.001	6.87 ± 6.12	0.007	5.55 ± 3.62	<0.001
LEV saliva concentration, μg/mL	10.69 ± 9.67	11.46 ± 10.1	6.97 ± 5.89	<0.001	7.13 ± 5.22	<0.001	7.2 ± 7.18	0.04	6.21 ± 4.99	0.054
Ratio saliva: plasma	0.99 ± 0.39	0.98 ± 0.4	1.02 ± 0.35	0.472	0.98 ± 0.36	0.979	1 ± 0.34	0.787	1.12 ± 0.36	0.199
DNCp of LEV, ng/mL/mg	9.15 ± 6.59	9.74 ± 6.57	6.23 ± 5.91	<0.001	7.12 ± 5.44	0.034	6.09 ± 7.46	0.008	4.56 ± 2.97	<0.001
DNCs of LEV, ng/mL/mg	8.96 ± 7.27	9.53 ± 7.39	6.18 ± 5.98	<0.001	6.65 ± 4.61	0.037	6.2 ± 7.93	0.031	5.14 ± 4.65	0.028
Only morning (trough steady‐state) saliva and plasma samples collected in pairs										
Sample pairs, No	318	258	60		25		22		13	
Seizure frequency in the last months prior to sampling	1.68 ± 3.68	1.54 ± 2.74	2.18 ± 5.89	0.278	2.29 ± 6.49	0.608	2.52 ± 6.54	0.502	1.11 ± 1.69	0.644
Seizure frequency in the 3 months prior to sampling	4.51 ± 9.61	4.33 ± 8.19	5.34 ± 14.62	0.516	4.58 ± 10.51	0.903	7.2 ± 20.02	0.532	2.5 ± 3.46	0.529
LEV daily dose, mg	1219.6 ± 503	1216 ± 494.7	1233 ± 540.5	0.814	1070 ± 364.6	0.151	1341 ± 638.8	0.27	1365 ± 600.5	0.295
LEV plasma concentration, μg/mL	7.85 ± 5.07	8.43 ± 5.18	5.36 ± 3.63	<0.001	5.48 ± 3.11	0.006	5.21 ± 4.23	0.005	5.4 ± 3.72	0.039
LEV saliva concentration, μg/mL	7.69 ± 5.83	8.18 ± 5.98	5.59 ± 4.63	0.002	5.59 ± 3.99	0.035	5.33 ± 5.17	0.021	6.01 ± 5.13	0.2
Ratio saliva: plasma	0.99 ± 0.41	0.98 ± 0.41	1.02 ± 0.37	0.441	1 ± 0.39	0.749	0.99 ± 0.35	0.909	1.12 ± 0.37	0.238
DNCp of LEV, ng/mL/mg	6.72 ± 4.16	7.24 ± 4.26	4.51 ± 2.83	<0.001	5.3 ± 3.05	0.027	3.71 ± 2.32	<0.001	4.34 ± 2.96	0.016
DNCs of LEV, ng/mL/mg	6.61 ± 4.94	7.08 ± 5.12	4.56 ± 3.45	<0.001	5.25 ± 3.42	0.081	3.61 ± 2.43	0.002	4.86 ± 4.71	0.126

Abbreviations: DNCp, dose‐normalized concentration in plasma; DNCs, dose‐normalized concentration in saliva; LEV, levetiracetam; SD, standard deviation.

### 3.2 Method validation

The calibration standard curves for levetiracetam were linear in the range of 0.1–50 mg/L in plasma and saliva (Table S1). All intra‐ and inter‐batch precision and accuracy values met the specified acceptance criteria (Table S2). There were no significant matrix effects in plasma and saliva. The results of recovery and matrix effects were shown in Table S3. Stability testing was designed to cover the anticipated conditions the patient samples may experience, showing that samples were stable at room temperature for 12 h, five frozen (−40°C) thaw cycles, five frozen (−80°C) thaw cycles, autosampler (15°C) for 24 h, 6 repeat injections, and 10‐fold dilutions (Table S4).

### 3.3 Correlation of levetiracetam concentrations between saliva and plasma

**FIGURE 1:**
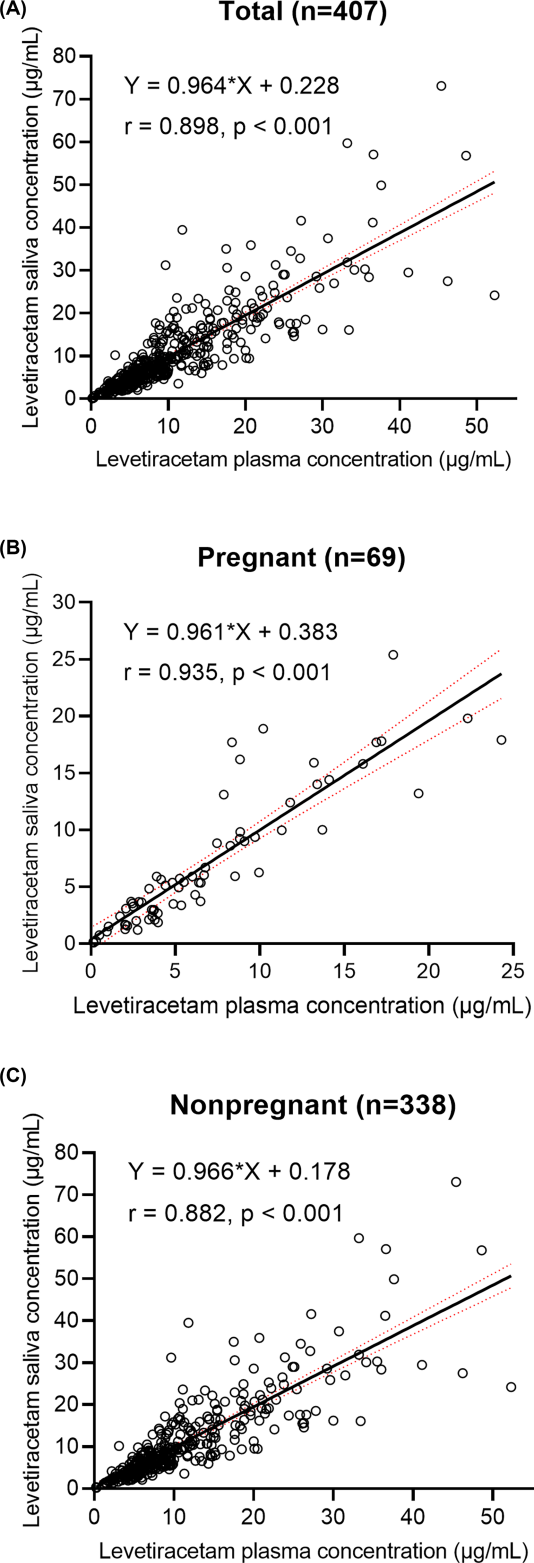
Relationship between measured plasma and saliva levetiracetam (LEV) concentrations in paired samples from (A) women with epilepsy, including (B) pregnant and (C) nonpregnant participants. Red lines indicate standard errors. The equation of the black lines refer to Pearson linear regression.

**FIGURE 2:**
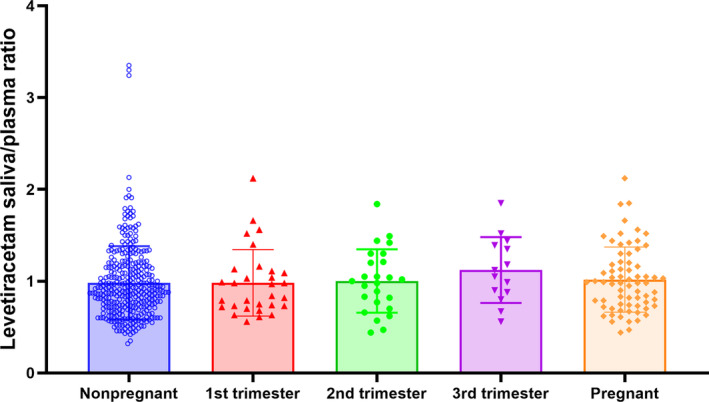
The ratio of saliva to plasma levetiracetam concentrations.

Spearman correlations displayed a good correlation between salivary and plasma levetiracetam concentrations were reported in the enrolled women with epilepsy (*r* = 0.898, *n* = 407, *p* < 0.001), including pregnant (*r* = 0.935, *n* = 69, *p* < 0.001) and nonpregnant participants (*r* = 0.882, *n* = 338, *p* < 0.001). Further regression analysis revealed that the concentration of saliva was linearly related to the concentration of plasma in all groups (Figure 1). Plasma concentrations were moderately higher than saliva concentrations, with ratios of saliva to plasma concentrations of 0.98 for nonpregnant women, 0.98, 1, and 1.12 for pregnant women during the first trimester, the second trimester, and the third trimester, respectively (Table 1, Figure 2).

**FIGURE 3:**
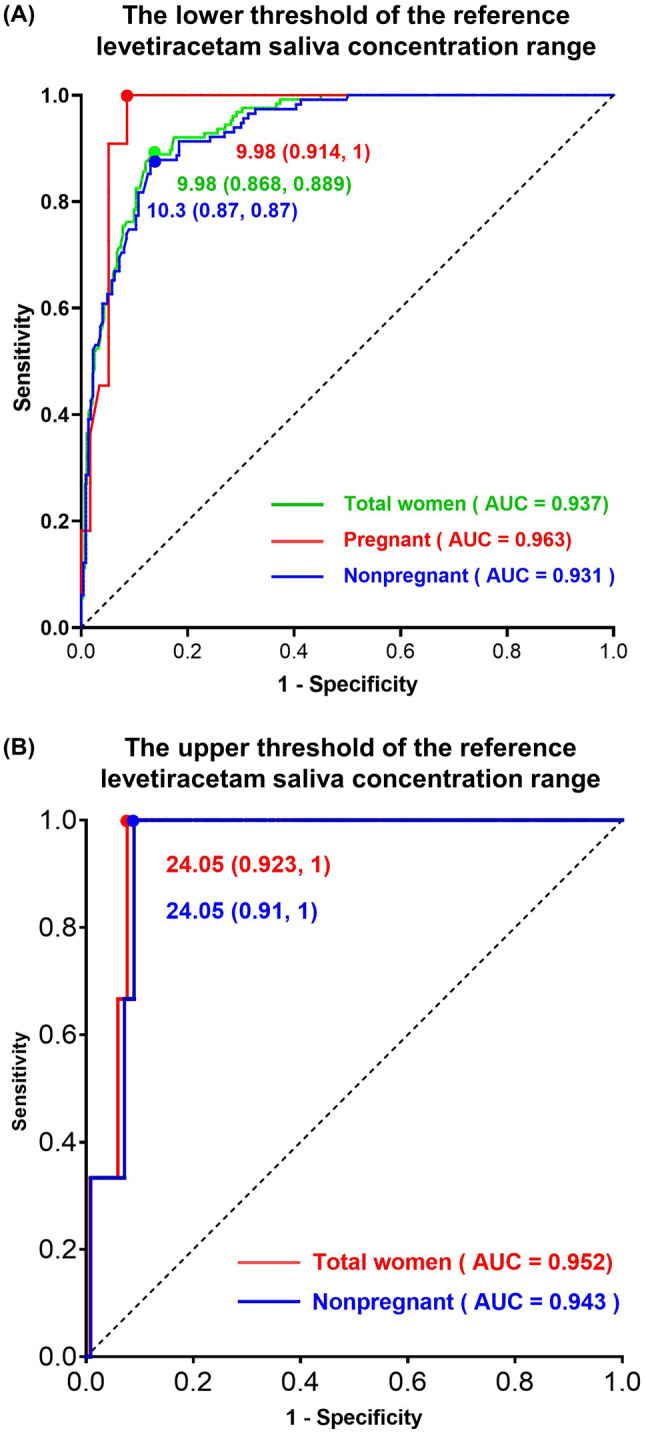
The Receiver Operating Characteristic (ROC) curve of levetiracetam concentration in saliva. The (A) lower and (B) upper thresholds of the reference level of levetiracetam concentration in saliva.

According to the recognized reference range of plasma levetiracetam concentration (12–46 μg/mL), among the 407 samples we tested, there were 126 plasma concentrations collected above 12 μg/mL that reached the minimum effective concentration. By plotting the ROC curve, we demonstrated that when the lower limit effective cutoff value for saliva concentration was 9.98 μg/mL, the area under the curve (AUC) for accurately identifying whether patients reached the minimum effective concentration through saliva monitoring was 0.937 (95% confidence intervals, 95% CI: 0.915–0.959), with a sensitivity of 88.9% and specificity of 86.8% (*p* < 0.001) (Figure 3A). In pregnant women, when using 9.98 μg/mL as the lower limit for saliva's minimum effective concentration, the accuracy of TDM testing was higher, with an AUC of 0.963 (95% CI: 0.921–1), sensitivity of 100%, and specificity of 91.4% (*p* < 0.001) (Figure 3A). Considering the upper limit of the reference concentration of levetiracetam plasma concentration as 46 μg/mL, we found three samples with plasma concentrations that exceeded the upper limit, and the patients were all in the non‐pregnant status during sampling. Statistical analysis revealed that when the cutoff value for saliva was 24.05 μg/mL, saliva TDM could accurately identify whether patients had concentrations that were too high and may produce toxicity, with an area under the curve of 0.952 (95% CI: 0.914–0.99), sensitivity of 100%, and specificity of 92.3% (*p* = 0.007) (Figure 3B).

### 3.4 Dose‐normalized levetiracetam concentration in plasma and saliva showed a similar trend of decreasing during the three trimesters of pregnancy

**FIGURE 4:**
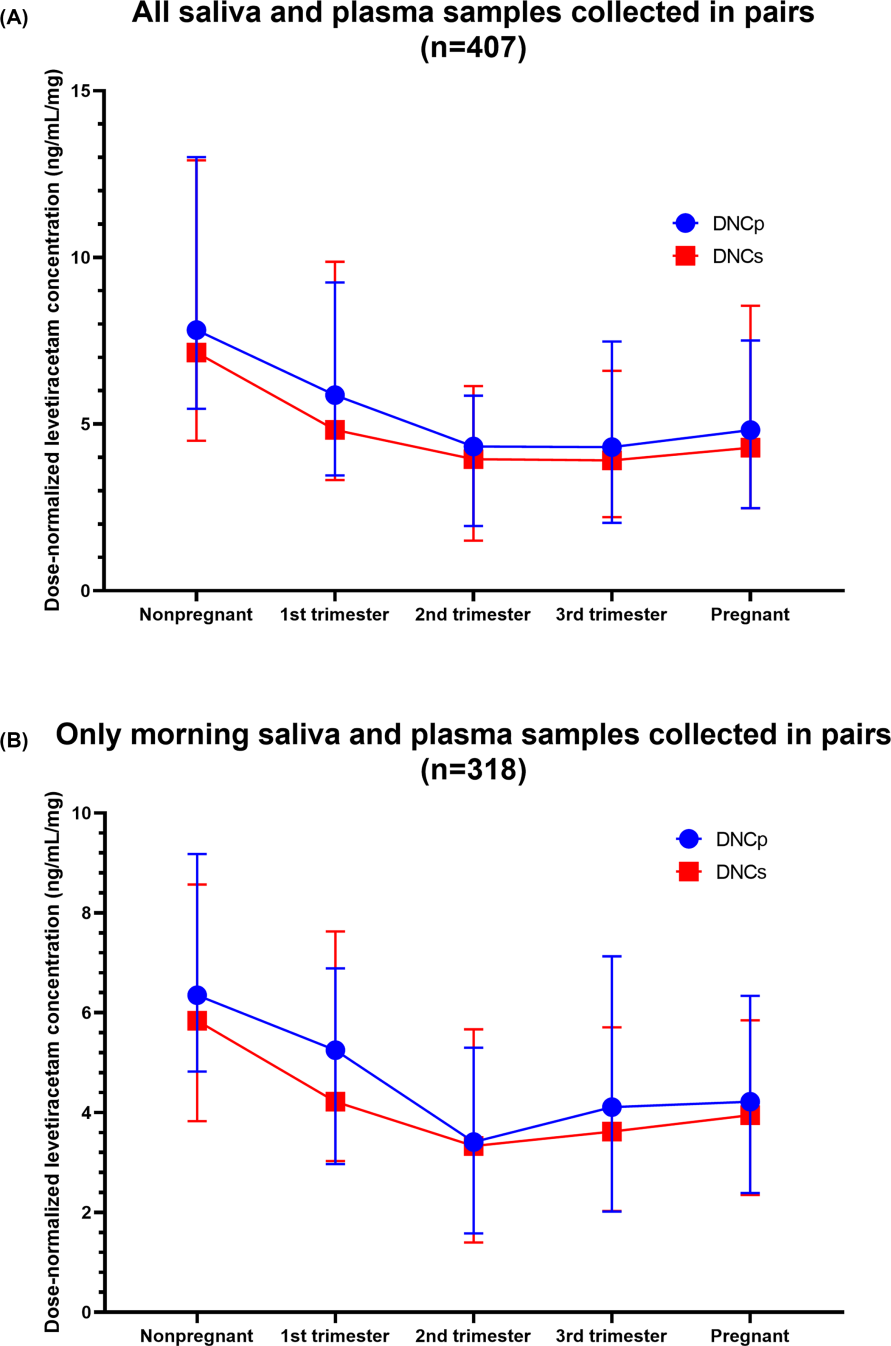
Dose‐normalized concentration in plasma (DNCp) and saliva (DNCs) in the three trimesters of pregnancy and nonpregnant women. (A) shows all saliva and plasma samples collected in pairs, including morning samples and samples taken after intake of levetiracetam; (B) shows only morning (trough steady‐state) saliva and plasma samples collected in pairs.

Compared with nonpregnant control participant values in morning samples and samples collected after intaking of levetiracetam, mean dose‐normalized concentration in plasma (DNCp) were significantly lower in pregnant women with a decrease of 36.0% (9.74–6.23 ng/mL/mg; *p* < 0.001), in the same way, saliva showed a similar trend of decreasing during the three trimesters of pregnancy with a 35.2% reduction (9.53–6.18 ng/mL/mg; *p* < 0.001) (Figure 4). As for morning (trough steady‐state) saliva and plasma samples collected in pairs, dose‐normalized levetiracetam mean trough concentrations during pregnancy were similarly decreased by up to 37.7% for plasma (7.24–4.51 ng/mL/mg; *p* < 0.001) and 35.6% for saliva (7.08–4.56 ng/mL/mg; *p* < 0.001), reaching their lowest levels in the second trimester, suggesting that TDM should be routinely performed during pregnancy. The detailed results are shown in Table 1.

## 4 DISCUSSION

To the best of our knowledge, this is the first study to explore the relationship between saliva and plasma concentrations of levetiracetam in Chinese women with epilepsy during pregnancy. We found a linear relationship between saliva and plasma levetiracetam levels, and the ratio of saliva to plasma levetiracetam concentrations was close to 1 and remained stable during pregnancy. This supports the use of saliva as a substitute for blood in monthly monitoring of levetiracetam concentrations during pregnancy, and this method should be widely promoted.

In this study, there was a significant correlation between plasma and saliva concentrations of levetiracetam (*r* = 0.898) among women with epilepsy. Interestingly, further analysis reveals that this strong correlation existed in both pregnant (*r* = 0.935) and non‐pregnant (*r* = 0.882) individuals, and the correlation was consistent with previous reports in the general population in Poland (*r* = 0.93)^19^ and Italy (*r* = 0.9).^22^ Additionally, the saliva‐to‐plasma concentration ratio of levetiracetam in all female epilepsy patients in this study was 0.99, which is consistent with the reported ratio of (0.73–1.34) in the general population.^19^, ^20^, ^22^ Interestingly, the saliva‐to‐plasma concentration ratio of levetiracetam in female epilepsy patients during the first, the second, and the third trimesters did not change significantly compared to non‐pregnant periods (*p* > 0.05), and remained stable at the range of 0.98–1.12. This indicates that the concentration of levetiracetam in saliva of female epilepsy patients during pregnancy is equivalent to that in plasma, and saliva monitoring results can replace plasma results.^9^

Dose‐normalized concentration in plasma and saliva underwent significant decreases during pregnancy (36%), which is consistent with previous study (36.8%).^7^ Free plasma concentration is more important than total plasma concentration because only free ASMs that cross the blood–brain barrier are considered to have pharmacological effects; however, it is more difficult to detect as it requires additional protein filtration procedures.^25^, ^28^, ^29^ Therefore, easily measurable saliva concentration reflecting the free drug concentration has a wide range of applications. In this study, saliva concentrations were found to be slightly lower than total plasma concentrations, which may be related to factors affecting drug concentration in saliva, such as the molecular mass of the drug, the lipid solubility characteristics of the drug, saliva pH, flow rate, and metabolism.^30^

The sample size of levetiracetam during pregnancy was small and a larger sample size will be needed in future studies. Nowadays, internationally used effective concentration ranges are based on total concentrations in serum and cannot be used for saliva monitoring. Our further study is establishing and evaluating a reference range for levetiracetam in saliva and the clinicians will be able to relate the answer from the laboratory to previous samples nor to reference ranges. Furthermore, there are any factors that may influence the salivary concentration of levetiracetam, such as food intake, oral hygiene, or diurnal variation, which will be investigated in our further studies.

## 5 CONCLUSION

In conclusion, salivary concentrations of levetiracetam are closely correlated with plasma levels and therefore saliva can be used as an alternative matrix to plasma for the concentration assay of the second‐generation antiseizure medication. The saliva concentration monitoring method established in our study could be further extended to facilitate telemedicine by collecting saliva samples at home, especially for the elderly and pregnant women with limited mobility.

## AUTHOR CONTRIBUTIONS

Wanling Li: Conceptualization, Investigation, Validation, Formal analysis, Visualization, Writing—original draft. Ximeng Yang: Data curation, Resources, Project administration. Qian Chen: Formal analysis. Zhenlei Wang: Methodology, Formal analysis. Yifei Duan: Data curation. Lei Chen: Supervision, Funding acquisition, Writing—review and editing. All authors read and approved the final manuscript.

## CONFLICT OF INTEREST STATEMENT

None of the authors has any conflict of interest to disclose. We confirm that we have read the Journal's position on issues involved in ethical publication and affirm that this report is consistent with those guidelines.

## Supporting information


Data S1.

## Data Availability

The data that support the findings of this study are available from the corresponding author upon reasonable request.
